# The Segmentation of Sub-Lexical Morphemes in English-Learning 15-Month-Olds

**DOI:** 10.3389/fpsyg.2013.00024

**Published:** 2013-02-06

**Authors:** Toben H. Mintz

**Affiliations:** ^1^Department of Psychology, University of Southern CaliforniaLos Angeles, CA, USA; ^2^Department of Linguistics, University of Southern CaliforniaLos Angeles, CA, USA

**Keywords:** language acquisition, morphology, infancy, speech perception, lexicon, psycholinguistics

## Abstract

In most human languages, important components of linguistic structure are carried by affixes, also called bound morphemes. The affixes in a language comprise a relatively small but frequently occurring set of forms that surface as parts of words, but never occur without a stem. They combine productively with word stems and other grammatical entities in systematic and predictable ways. For example, the English suffix -*ing* occurs on verb stems, and in combination with a form of the auxiliary verb *be*, marks the verb with progressive aspect (e.g., *was walking*). In acquiring a language, learners must acquire rules of combination for affixes. However, prior to learning these combinatorial rules, learners are faced with discovering what the sub-lexical forms are over which the rules operate. That is, they have to discover the bound morphemes themselves. It is not known when English-learners begin to analyze words into morphological units. Previous research with learners of English found evidence that 18-month-olds have started to learn the combinatorial rules involving bound morphemes, and that 15-month-olds have not. However, it is not known whether 15-month-olds nevertheless represent the morphemes as distinct entities. This present study demonstrates that when 15-month-olds process words that end in *-ing*, they segment the suffix from the word, but they do not do so with endings that are not morphemes. Eight-month olds do not show this capacity. Thus, 15-month-olds have already started to identify bound morphemes and actively use them in processing speech.

## Introduction

In most human languages important components of linguistic structure are carried by affixes, or bound morphemes. The affixes in a language comprise a relatively small but frequently occurring set of forms that surface as parts of words, but never occur without a stem. While bound morphemes always occur as part of a larger word, they are viewed as having an independent status by virtue of the fact that they combine productively with stems and other grammatical elements in systematic and predictable ways. For example, any English verb root that is inflected with the suffix *-ing* and is preceded by a form of the auxiliary verb, *be*, results in a verb form that is marked with particular tense and aspect: present progressive (e.g., *she is reading*). Mastering the morphological system of a language thus involves acquiring the generalizations about the relationships between formal elements (e.g., auxiliary-*be* and *-ing*), as well as the semantic and functional properties of the language that are represented in the morphological system (e.g., mood, aspect, number, etc.). However, before learners can acquire morphological facts about their language, they must first identify the sub-lexical combinatorial units: they must identify the bound morphemes.

Children’s first productive use of bound morphemes (and functional categories more broadly, including function words) is delayed relative to their initial production of content words. For example, children typically produce their first words at approximately 12 months, but it is not until they combine words, between 18 and 24 months, that children learning English begin to produce morphemes when they are required (Brown, 1973; de Villiers and de Villiers, 1973), and even then, mastery may be limited to a small number of forms.

From perception and comprehension studies, there is also evidence that infants learning English have started to form representations of sub-lexical morphemes, and have learned something about the patterns in which the morphemes normally occur, by the time they start producing two-word combinations (Santelmann and Jusczyk, 1998; Golinkoff et al., 2001; Soderstrom et al., 2002). For example, Santelmann and Jusczyk (1998) showed that 18-month-old infants preferred to listen to grammatical sentences in which a word ending in the morpheme *-ing* followed the function word *is* (1a), over ungrammatical sentences in which the word followed the function word *can* (1b).

(1) a. At the bakery, everybody is baking bread.
b. *At the bakery, everybody can baking bread.

However, Santelmann and Jusczyk did not find such a differential preference in 15-month-olds. Similarly, for the inflection *-s* (plural and third person singular), Soderstrom (2003) and Soderstrom et al. (2002) showed that 19-month-olds noticed when normal dependencies between the affix and nearby function words were violated, but 16-month-olds did not. However, Soderstrom et al. (2007) reported some conditions under which even 16-month-olds show a sensitivity to a misplaced *-s* affix. Taken together, these experiments demonstrate that by 18 months, English-learning infants have learned morphosyntactic patterns involving a range of sub-lexical morphemes, and suggest that infants’ sensitivity to some of these patterns is developing at 16 months. As a consequence, these studies also provide evidence concerning when learners represent affixes as distinct forms – that is, separate from the stems to which they are attached – since infants must first segment the affixes as distinct units before learning patterns to which they contribute.

Similar experiments with infants learning German (Höhle et al., 2006), Dutch (van Heugten and Johnson, 2010), and French (van Heugten and Shi, 2010; Nazzi et al., 2011) have broadly replicated the finding that infants between 17 and 24 months are becoming sensitive to morphosyntactic patterns involving affixes, and to functional elements more broadly (van Heugten and Shi, 2009; Shi and Melançon, 2010). At the same time, these cross-linguistic studies provided further insights into the distributional and linguistic factors that influence how infants process morphosyntactic dependencies. However, these studies leave open the question of infants’ representations of sub-lexical morphemes in the developmental period before they show sensitivity to dependencies between morphosyntactic units. That is, it is not clear precisely *why* 15-month-olds failed to respond differently to (1a) and (1b) in Santelmann and Jusczyk’s (1998) study. There is evidence that between 11 and 14 months, infants acquire representations of function words (Shi et al., 2006a,b), so 15-month-olds’ behavior is not likely to be due to an inability to distinguish *is* in (1a) from *can* in (1b). However, it could be that 15-month-olds simply do not represent *-ing* as a discrete unit, and therefore have no way of representing patterns and dependencies involving that morpheme. On the other hand, they might have a discrete representation of *-ing*, but have not yet learned the dependency patterns in which *-ing* participates. Resolving this question is important for understanding the time-course of infants’ morphosyntactic development, as well as for providing a basis for further research into the mechanisms of infants’ morphosyntactic acquisition.

A recent study of French-learning infants is relevant to this question. Marquis and Shi (2012) familiarized French-learning 11-month-olds to a pseudo-root (i.e., a nonsense syllable). They then recorded infants’ listening times to passages containing the pseudo-root “inflected” with the actual French suffix, /e/, and to sentences with an unfamiliarized pseudo-root, also ending in /e/. Infants listened longer to the sentences containing the inflected familiarized pseudo-root, suggesting that infants segmented the /e/ ending from the rest of the word and recognized the familiar stem. Different infants who were tested on familiarized and unfamliarized pseudo-roots inflected with /u/, which is not a French affix, did not listen preferentially to either stimulus type. Thus, the response of infants who preferred the familiarized vs. unfamiliarized stems with the /e/ suffix cannot be attributed to phonetic similarity of the familiarized and tested forms; rather, infants’ behavior was apparently guided by factors relating to the status of /e/ as a morpheme. Marquis and Shi’s study provides the earliest evidence for infants’ segmentation of sub-lexical morphemes.

Marquis and Shi’s (2012) results demonstrate that infants have begun to form representations of bound morphemes by the end of the first year of life, at least in the case of infants learning French. In considering the question of English-learners’ representation of *-ing*, it is tempting to extend this finding to English, and conclude that English 15-month-olds must therefore represent *-ing* as a discrete form. However, there are important differences between French and English that might affect how Marquis and Shi’s conclusions from French generalize to English. Foremost is that the inflectional system of French is overall richer than that of English. French marks both grammatical gender and number, and has gender and number agreement between nouns, pronouns, determiners, and adjectives. These properties might lead French-learning infants to attend to, detect, and process suffixes at an earlier age compared to infants learning English and other languages in which overt morphology is relatively impoverished. It is therefore important to verify the finding in other languages.

There are also methodological considerations that limit the generalizability of Marquis and Shi’s (2012) findings. In their experiments, infants were familiarized to a pre-segmented stem, and only had to process and recognize that stem in combination with a suffix. If infants’ early representations of sub-lexical forms are fragile, their ability to detect and process bound morphemes may be limited. The processing demands of tracking one pre-segmented stem over the course of an experiment may be simple enough for detection of the morpheme and subsequent segmentation of the stem, but sub-lexical processing could be hindered in more complex situations. Replicating the finding with different experimental designs, especially those that place more demands on processing and memory resources, is important for establishing the robustness of infants’ early representations of morphology. In each experiment in the current study, infants were exposed to a multitude of stems inflected with *-ing*. In order to show evidence of morphological segmentation they had to segment the stems from these forms, remember them over the course of the familiarization period, and then recognize them during the test trials. While infants would not need to segment and retain every stem in order to show a reliable segmentation effect, they would have to track several, thus increasing complexity and resource demands. Furthermore, requiring infants to perform the segmentation during the familiarization phase rather than at test – reversing the method of Marquis and Shi – could increases task difficulty as well. When the bare stem is given first it can aid infants in detecting the relevant words in the test passages, making the task of detecting the stem in the inflected form somewhat easier. However, when the inflected forms are given first (particularly when they are in passages, as in Experiments 2–4), infants do not have this extra guide to morphological segmentation.

In summary, Marquis and Shi’s (2012) findings provide important evidence that infants can represent sub-lexical morphemes well in advance of their ability to track the dependency patterns in which they occur. However, typological differences between English and French, as well as the single methodological context of the findings only provide indirect evidence with respect to morphological representations in English-learners. Thus, the question of whether English-learning 15-month-olds treat *-ing* as a distinct form [and, thus, their apparent insensitivity to the violation in (1b)] remains open. The present study provides a more direct assessment of English-learning 15-month-olds’ morphological representations. Experiments 1–3 use multiple designs and stimuli sets to provide converging evidence that English-learning 15-month-olds treat *-ing* as a distinct unit. Evidence for a discrete representation is inferred from infants’ ability to segment *-ing*, in contrast to non-morpheme suffixes, from the ends of novel words. Motivated by the formal similarities of sub-lexical segmentation and word segmentation, Experiment 4 goes on to test for evidence of sub-lexical segmentation in 8-month-olds, who have been shown to segment words from continuous speech (Jusczyk and Aslin, 1995; Saffran et al., 1996; Jusczyk et al., 1999; Pelucchi et al., 2009).

## Experiment 1

This experiment tested the hypothesis that English-learning 15-month-olds represent the suffix -*ing* as a distinct entity, and that the representation as a distinct form influences infants’ parsing and representation of words.

Infants were familiarized to novel words, spoken in isolation. Some of the words ended in the English morpheme, *-ing* (e.g., *lerjoving*), and others ended with the phoneme sequence /ɑt/ (*-ot*, e.g., *jemontot*), while others did not systematically share an ending. The prediction was that if 15-month-olds represented the suffix *-ing* as a distinct entity, then they would be more likely to segment *-ing* from the ends of the novel words than they would the pseudo-suffix *-ot*. As a consequence of the segmentation process, infants would then store a representation of the resulting isolated novel “stems” (*-ing stems*, e.g., *lerjov*, in the example above). Since, by hypothesis, infants would not perform this kind of sub-lexical segmentation with words ending in *-ot* (or would be considerably less likely to), they should not form sub-lexical representations of the stems of words ending in *-ot* (*-ot stems*). As a result, infants should find *-ing* stems more familiar than *-ot* stems after familiarization. Differences in responses were tested using a version of the Head-turn Preference Procedure (HPP; Kemler Nelson et al., 1995).

### Materials and methods

#### Subjects

All experiments reported in this paper were approved by the University of Southern California’s Institutional Review Board. Subjects were recruited by telephone from a database of parents who had expressed interest in having their infant participate in research in our lab. At least one parent of each infant provided informed consent before the infant participated in the experiment. At the conclusion of each test session, we gave the parent a t-shirt for their child that read, “Graduate of the University of Southern California Language Development Lab,” as a token of our appreciation.

Data for 24 English-learning 15-month-olds were analyzed (mean age 14:25, range 14:15–15:10). An additional 15 infants were tested but were excluded from the data analysis due to failure to complete the experiment (6), failure to attend for more than 1 s to at least three test trials per block (5), excessive fussiness (2), parental interference (1), infant moved out of view (1). Twelve subjects were randomly assigned to familiarization group A; the remaining 12 were assigned to familiarization group B.

#### Stimuli and design

Familiarization and test stimuli were recorded by a female, native American English speaker, who was blind to the purpose of the study. Recordings were made in a sound attenuating booth, using a Shure SM58 microphone. Stimuli were digitized directly to a computer, at a sampling rate of 44.1 kHz. Three instances of each of the familiarization and test items were recorded. All stimuli were recorded during the same recording session.

##### Familiarization stimuli

Familiarization stimuli consisted of two sets, A and B, each consisting of 16 nonce words. In each set, five words ended in the English suffix *-ing*, five ended in the non-morphological ending *-ot* (/ɑt/), and the remaining six words were “uninflected” – that is, ending in a phoneme sequence that was not shared by other familiarization words. The goal in including the uninflected fillers was to add some variety to the familiarization material to help maintain infants’ engagement in the experiment. With respect to the design of the experiment, words ending in *-ing* and *-ot* were treated as a pseudo-stem plus an *-ing* or -*ot* suffix. Pseudo-stems in *-ing* words are called*-ing stems* and pseudo-stems in *-ot* words are called *-ot stems*. Sets A and B were designed to counterbalance stems and endings, such that *-ing* stems in one set were *-ot* stems in the other set. The “uninflected” words in both sets were the same. Table 1 shows the complete set of familiarization stimuli for Experiment 1.

**Table 1:** **Familiarization material for experiment 1**.

Set A			Set B		
Gorping	Rimpot	Choon	Gorpot	Rimping	Choon
Feming	Genot	Wug	Femot	Gening	Wug
Fejing	Sibot	Zimp	Fejot	Sibbing	Zimp
Gemónting	Jivántot	Pux	Gemóntot	Jivánting	Pux
Lérjoving	Káfteeot	Grífdon	Lérjovot	Káfteeing	Grífdon
		Bincáde			Bincáde

*Half the subjects heard Set A, half heard Set B. For bisyllabic words, the stressed syllable is indicated with the accent mark*.

Four of the pseudo-stems were bisyllabic and the remainder were monosyllabic. Stem length was included as a variable in order to increase the variety of the familiarization material, and also to investigate the influence of word complexity on infants’ ability to detect suffixes. For bisyllabic stems, stress was controlled such that trochaic and iambic stems occurred equally often with *-ing* and *-ot* endings (see Table 1).

##### Test stimuli

Test stimuli consisted of the 10 pseudo-stems that were “inflected” in the familiarization sets, but now without the suffixes (e.g., *gorp, rimp, gemónt*, etc.). There were four unique test stem types, characterized by their value on two dimensions: number of syllables, and stem status. Stems were either monosyllabic or bisyllabic (derived from bisyllabic and trisyllabic familiarization words, respectively), and were either *-ing* stems or *-ot* stems. While the test stimuli were identical for all infants, the status of the stem – that is, whether it was an *-ing* stem or *-ot* stem – depended on the infant’s familiarization set. This design feature counterbalanced stem status for each test stem. Table 2 shows the test stems, organized by number of syllables and stem status.

**Table 2:** **Test stimuli for experiment 1**.

**MONOSYLLABIC TRIALS**		
*-ing* stems for group A	*Gorp, fem, fej*	*-ot* stems for group B
*-ot* stems for group A	*Rimp, gen, sib*	*-ing* stems for group B
**BISYLLABIC TRIALS**		
*-ing* stems for group A	*Gemónt, lérjov*	*-ot* stems for group B
*-ot* stems for group A	*Jivánt, káftee*	*-ing* stems for group B

*Each row specifies the stems used in one test trial*.

##### Acoustic properties

**Figure 1:**
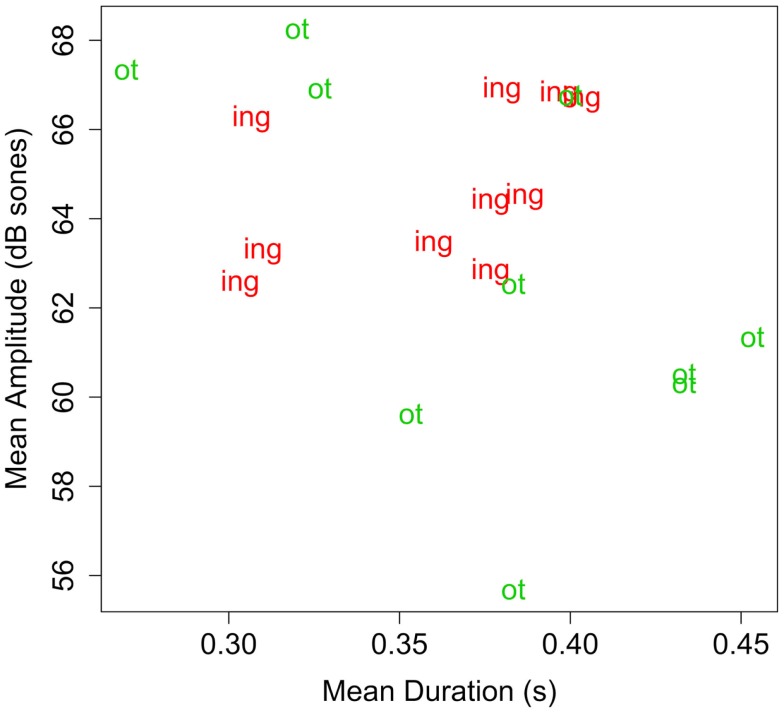
**Plot of duration (s) by amplitude (dB sones) for suffixes in Experiment 1**. Each data point represents the mean of the duration and amplitude of the affix, averaged across the three tokens of a familiarization word.

To ensure that any differences in infants’ ability to segment *-ing* and *-ot* could not be due to acoustic differences between the endings, the mean amplitude and duration of *-ing* and *-ot* in tokens of the familiarization materials were measured using Praat (Boersma and Weenink, 2009). Since each word was realized in three tokens, acoustic measures were averaged across the three tokens for each word. Table 3 presents the mean values for each suffix, organized by word stem. Figure 1 depicts these means graphically, indicating the affix type. As the table and figure show, the endings are not systematically different as a function of either dimension nor simple combination of dimensions.

**Table 3:** **Measurements of duration and intensity of the English affix and pseudo-affix used in Experiment 1**.

	Duration (s)		Amplitude (dB sones)	
Stem	-ing	-ot	-ing	-ot
Fej	0.36	0.27	63.44	67.34
Fem	0.39	0.45	64.50	61.35
Gemont	0.31	0.32	63.28	68.26
Gen	0.31	0.38	66.24	55.69
Gorp	0.38	0.33	66.89	66.92
Jivant	0.38	0.43	64.39	60.32
Kaftee	0.38	0.43	62.81	60.52
Lerjov	0.30	0.40	62.55	66.77
Rimp	0.40	0.35	66.80	59.63
Sib	0.40	0.38	66.68	62.54
Mean	0.36	0.38	64.76	62.93

*For each row, measurements were averaged from the three recorded tokens of the relevant word form (ending in *-ing* or *-ot*)*.

#### Procedure and apparatus

Each infant was tested separately while seated on a caretaker’s lap in the center of a sound-attenuated room. The caretaker listened to masking music over close-fitting headphones, in order not to hear the experimental material. An experimenter observed the infant’s looking behavior through a closed-circuit television monitor in an adjacent room. The experimenter registered the infant’s head-turn responses into a computer that controlled all aspects of the experiment.

At the start of the familiarization phase, a red light positioned at eye level on the wall directly in front of the infant flashed repeatedly. When the infant oriented toward the light, the familiarization material was played on two loudspeakers mounted on the walls to the left and right of the infant. When the familiarization stream started, the center light was extinguished and a light mounted above one of the loudspeakers flashed. It continued to flash until the infant first looked toward it, then looked away for two consecutive seconds. The side light was then extinguished and the center light flashed again until the infant oriented to the neutral center position. This process was repeated for the duration of this phase, randomizing the side on which the light flashed. The interactions with the lights kept the infants engaged, and established the contingency between their looking behavior and the activation of the lights.

The familiarization material played continuously, during the entire familiarization phase, and was not dependent on the infants’ orientation once the trial began. The 16 familiarization words were presented in five blocks, with the order of words randomized within each block, and with a different random order for each infant. There was a 300 ms silence between each word. Since there were three recorded versions of each word (see section Stimuli and Design), the computer randomly selected one of the three tokens on each presentation. Half the subjects heard word set A words, and the other half heard set B words. The total familiarization period lasted approximately 80 s.

A brief contingency training phase immediately followed the familiarization phase. Here, presentation of the auditory stimuli was also contingent on the infant orienting to the flashing side light. The auditory stimulus was always a 440 Hz pure tone lasting 1 s. Presentation started when the infant oriented toward the flashing side light, and the tone was repeated until the infant looked away for two contiguous seconds. This phase consisted of four such trials. Its purpose was to prepare the infant for the test phase that immediately followed, in which auditory stimulus presentation was similarly contingent on orienting to the flashing light.

The test phase was similar to the contingency training phase except that in each test trial, a sequence of stems was played. Table 2 shows the four trial types that determined which particular sequences of stems was played. Trial types were defined by the length in syllables of the stems, and the ending that was associated with the stems during familiarization. Stems were played in the order shown, with and ISI of 300 ms. The sequence was repeated within a test trial until the infant looked away for two consecutive seconds, or after 15 repetitions of the sequence. Test trials were presented in two blocks, with trial order randomized within blocks, for a total of eight test trials per infant. The computer recorded the duration of each trial. The progression from one trial to the next was no different for trials within a block compared to the transitions from the first to the second block.

In all phases of the experiment the stimulus presentation side on a given trial was randomly selected. However, the selection was constrained such that stimuli would not be presented to the same side in more than three consecutive trials.

If infants segment the suffix *-ing* from familiarization words, then the *-ing* stems should be relatively familiar to them, as they are an outcome of the segmentation process. If infants do not segment the pseudo-suffix, *-ot*, then the *-ot* stems should be relatively less familiar. Differences in familiarity are predicted to result in differences in listening times to the two types of stimuli.

### Results

Listening times under 1 s were replaced with the listening time for the same stimuli in the alternate block. This criterion was used to identify trials in which infants looked away before they heard at least one entire stem in the trial, as such trials were not thought to be informative about the representations of interest. This resulted in one replacement for a bisyllabic *-ot* stem trial, and one replacement for a monosyllabic *-ing* stem trial. However, as described in the subject selection section, infants who maintained a head-turn for less than 1 s on more than one trial per block were not included in the data analysis.

The data were first submitted to an analysis of variance (ANOVA) with stem type (*-ing* or *-ot*) and length in syllables (1 or 2) as within-subjects factors, and familiarization group (A or B) as a between subjects factor. Since there were no significant main effect or interactions involving familiarization group, all further analyses combined group A and B, to increase power. In the resulting 2 × 2 ANOVA, there were no main effects, but there was a significant interaction between stem type and number of syllables in the stem [*F*(1,23) = 4.47, *p* = 0.046].

**Figure 2:**
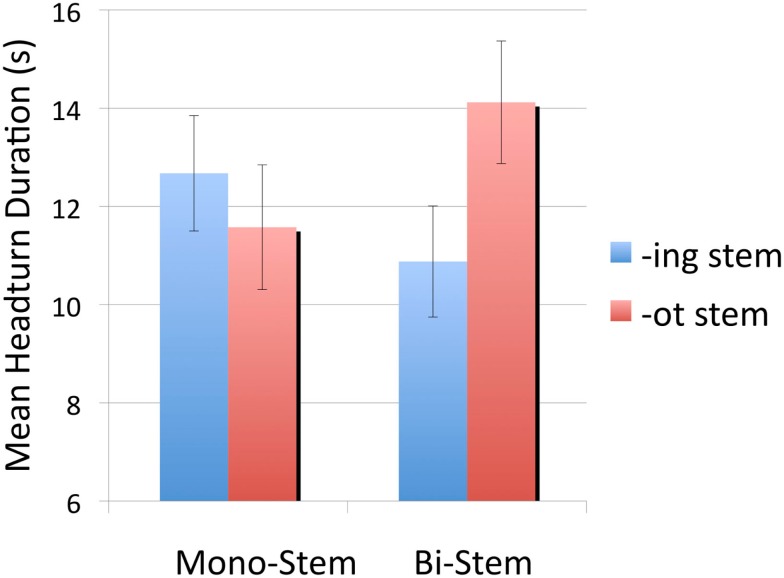
**Mean listening times for Experiment 1, organized by stem length in syllables and stem status**. Error bars show standard errors.

In order to understand this interaction, infants’ mean listening times to *-ing* stems and *-ot* stems were compared separately for monosyllabic and bisyllabic stems. For the monosyllabic stems, infants’ mean listening times to *-ing* and *-ot* stems were 12.70 s (SE = 1.18) and 11.60 s (SE = 1.27) respectively. A paired *t*-test showed that these listening times were not significantly different [*t*(23) = 0.79, *p* = 0.44]. However, for bisyllabic stems, infants listening significantly longer to *-ot* stems (*M* = 14.1s, SE = 1.3) compared to *-ing* stems [*M* = 10.9s, SE = 1.1;*t*(23) = 2.42, *p* = 0.024, *d* = 0.56]. Sixteen out of the 24 infants listened longer to bisyllabic *-ot* stems. Figure 2 depicts listening times to each stem type, organized by length in syllables.

### Discussion

Overall, this experiment provides evidence that by 15 months, English-learning infants treat*-ing* in a special way, such that when they hear a word that ends in that sequence, they segment it from the rest of the word. The evidence comes from comparing test trials in which subjects heard stems to which they were familiarized in words ending in *-ing* vs. words ending in *-ot*. When the stems were bisyllabic, subjects listened longer to the *-ot* stems. Under the assumption that infants had segmented the morphemic stems many times during familiarization, and thus experienced them as an entity distinct from the larger word, the listening differences are consistent with a novelty preference for the *-ot* stems, which, by hypothesis, the subjects had not previously segmented from the familiarization words.

It is not clear why such a difference was not observed for monosyllabic stems. One possibility is that the longer words were more salient in the familiarization phase, and were fore grounded against a background of shorter words. Infants may not have processed the words with monosyllabic stems to the same degree as the words with bisyllabic stems, and therefore may not have segmented either *-ing* or *-ot* from those words. In general, the variable length of the novel words might have disrupted infants ability to segment morphemes across all words (Johnson and Tyler, 2010), and the longer, trisyllabic words (i.e., with bisyllabic stems) may have been more effective in capturing infants’ attention^1^. Infants’ ability to segment *-ing* from monosyllabic stems is explored further in Experiment 3.

As the measurements graphed and shown in Figure 1 and Table 3 indicate, there are no obvious differences in acoustic salience could have influenced sub-lexical segmentation in a way that would have given rise to the observed results in Experiment 1. Nevertheless, it is worth replicating the finding with different stimuli. With this in mind, Experiment 2 replicates the general finding from Experiment 1 with a different pseudo-affix and a slightly modified design.

## Experiment 2

Experiment 1 provided evidence that is consistent with the interpretation that 15-month-olds preferentially segment *-ing* (as opposed to non-morphemic endings) from words, indicating that they represent *-ing* as a distinct entity. However, the experiment contrasted *-ing* with just one pseudo-affix, *-ot*. It is possible that *-ing* was intrinsically easier for infants to segment than *-ot*, although the acoustic measures do not support this possibility (see Table 3). Nevertheless, in order to be confident that the results were not due to some idiosyncratic property of *-ot*. Experiment 2 replicated the general design, but with the pseudo-affix *-dut*. The most obvious difference between the two pseudo-affixes is that *-dut* begins with a stop consonant, whereas *-ot* (like *-ing*) begins with a vowel. At a phonological level, the presence of an onset makes *-dut* more complete as a syllable, compared to *-ot* (and *-ing*), and therefore might increase the chances that the pseudo-suffix will be segmented from the rest of the word (Hayes, 2009). The acoustic properties of *-dut* and *-ing* in Experiment 2 are presented and discussed below.

To make the infants’ experience more like one in a normal language context, the familiarization material presented the novel words in English sentences – e.g., *I see you lérjoving!* – rather than in isolation as in Experiment 1. Situating the novel words in simple sentences made the familiarization stimuli more natural than a list of isolated words. The natural contexts could lead to a greater engagement of language processing mechanisms, for example, those involving word segmentation, syntactic and semantic processing. Detecting and segmenting sub-lexical forms might then be enhanced by greater overall linguistic processing. On the other hand, the natural contexts are also more complex, with more material to process in a given utterance, and a greater demand on resources (assuming that subjects are carrying out processing at these other linguistic levels to some degree). We might, hence, observe an advantage for sub-lexical segmentation of forms that are more familiar to infants based on their experience with English, such as the suffix *-ing*.

### Materials and methods

#### Subjects

Subject recruitment procedures were identical to those used in Experiment 1.

Thirty infants averaging 15 months of age participated in the experiment (mean age 15 months 3 days, range 14:15–15:18). Fifteen were randomly assigned to familiarization group A and the remaining subjects were assigned to familiarization group B. An additional 28 subjects were tested, but were excluded from the study due to failure to complete the experiment (15), failure to orient for at least 2 s in at least three trials per block (2), parental interference (3), excessive fussiness (6), equipment failure (1), and experimenter error (1).

#### Stimuli and design

The nonsense words were the trisyllabic words from Experiment 1. Each nonce word occurred in two different sentences, yielding a total of eight unique familiarization sentences. In all sentences, the nonce word was the final word in the sentence and was in the syntactic position of a verb. Two counterbalanced sets of familiarization sentences (set A and set B) were created. The sentences in set A are given in Table 4. Set B was created from set A by exchanging *-dut* and *-ing* endings on the nonce words in the sentences in Table 4. For example, the sentence *I see you lérjoving* in set A corresponded to *I see you lérjovdut* in set B.

**Table 4:** **Familiarization sentences for subjects in group A, in Experiment 2**.

Sentences with *-ing* words	Sentences with *-dut* words
I see you *lérjoving*!	Does Sam want to go *káfteedut*?
Johny likes *gemónting*!	I want to go *jivántdut*!
Do you want to go *lérjoving*?	Harold likes *káfteedut*!
Can you see me *gemónting*?	Can you see Sally *jivántdut*?

*Familiarization sentences for subjects in group B were identical, except that *-ing* stems and *-dut* stems were switched*.

The familiarization sentences were recorded by a female native English speaker, who was blind to the predictions of the experiment. The speaker was trained to produce the sentences with normal prosody that was appropriate for a simple declarative sentence or a question. The sentences were compiled into three lists, each listing the sentences in a different random order. The speaker was recorded reading each list, resulting in three separate instances of each familiarization sentence, from which the most natural sounding version was selected for use in the experiment.

Test items were the four bare nonce stems: *lérjov, gemónt*, *káftee, jivánt*. For a given subject, half the test stems were *-ing* stems, and half the stems were *-dut* stems. Due to the counterbalancing procedure, the *-ing* stems for subjects in group A were the *-dut* stems for subject in group B, and vice versa. Hence, any overall differences in infants’ responses to *-ing* stems and*-dut* stems could not to be due to idiosyncrasies of the test items themselves, but rather must be related to differences in the test items’ distribution in the familiarization strings.

Recall that the stress pattern was trochaic (strong-weak) for half of the nonce stems and iambic (weak-strong) for the other. Stress is known to be a factor in infant speech processing (Jusczyk et al., 1993; Echols et al., 1997; Thiessen and Saffran, 2003; Curtin et al., 2005; among others), and hence could influence sub-lexical segmentation. Consequently, stress pattern was incorporated as a controlled variable in the experimental design. The stress pattern for one nonce stem from each stem category (*-ing* and *-dut*) was trochaic and the other was iambic.

Test items were recorded by the same trained speaker who recorded the familiarization sentences. The stems were produced with list intonation, and each word was recorded three times and digitized onto the computer that controlled the experiment. When playing test items, the computer randomly selected one of the three instances of the item to play.

##### Acoustic properties

**Figure 3:**
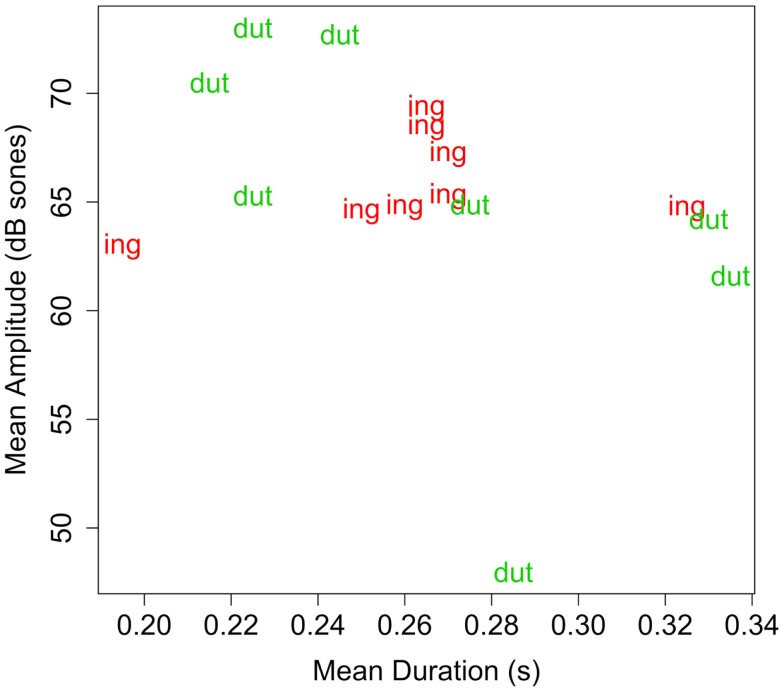
**Plot of duration (s) by amplitude (dB sones) for suffixes in Experiments 2–4**. Each data point represents the two measurements of the affix (of the type designated by the label) of a token of a familiarization word.

Although instances of *-ot* and *-ing* in Experiment 1 did not differ, overall, in the dimensions of intensity and duration (see Table 3), it is possible that some other factors made *-ot* particularly resistant to segmentation. The pseudo-affix used here, *-dut*, is more well-formed as a syllable than *-ot* due to the presence of an onset (Hayes, 2009), and should not be resistant to segmentation on phonological grounds. To compare acoustic intensity of -*dut* and *-ing*, the mean intensity for the two endings was measured in each familiarization sentence using the Praat software package (Boersma and Weenink, 2009). Each novel word occurred in two familiarization sentences, so measurements for each word were averaged across its two tokens. Table 5 reports these means for each word, and Figure 3 plots the endings on the two dimensions. (Items from Experiment 3 are also shown.) Clearly, on these acoustic measures, *-ing* and *-dut* are not systematically different. Thus, not only is the pseudo-suffix a CVC syllable, it is matched with *-ing* in duration and intensity. Thus, on acoustic-phonetic grounds, the pseudo-suffix should be just as easy to segment from the pseudo-stem as the actual English suffix.

**Table 5:** **Duration and intensity measurements for *-ing* and pseudo-suffixes on target words in Experiments 2–4**.

	Duration (s)		Amplitude (dB sones)	
Stem	-ing	-dut	-ing	-dut
Gemont	0.25	0.28	64.58	64.89
Jivant	0.26	0.33	64.78	64.22
Kaftee	0.33	0.34	64.72	61.61
Lerjov	0.27	0.29	65.27	47.98
Fem	0.20	0.25	62.96	72.72
Gorp	0.27	0.22	67.22	70.50
Riz	0.27	0.23	69.34	73.02
Mean	0.27	0.23	65.91	65.30

*Bisyllabic stems were used in Experiment 2 and 4, and monosyllabic stems were used in experiment 3. Values are averaged across the two tokens of each word*.

#### Procedure and apparatus

The apparatus that was used in Experiment 1 was used in Experiment 2, however the procedure varied in several ways. First, the familiarization stimuli were presented in six blocks, rather than five. Subjects thus heard an additional repetition of each novel word in this experiment. The total duration of the familiarization phase was approximately 90 s. Familiarization utterances were presented with an ISI of 200 ms.

The test phase also differed from Experiment 1 in that here, each test trial repeated only one stem, rather than multiple stems of the same type. Thus, there were four unique test trials, together constituting every combination of stem type (*-ing* vs. *-dut*) and stress pattern (trochaic vs. iambic). Due to the counterbalanced design, *-ing* stems for group A subjects were *-ot* stems for group B subjects, and vice versa. As in Experiment 1, test trials were presented in two blocks, with order randomized within blocks.

All other aspects of the procedure were identical to Experiment 1.

### Results and discussion

Test trials with a listening time under 1 s were replaced with the listening time for the same stimulus in the other block. Data for one *-ing* stem trial and one *-dut* stem trial were modified in this way.

**Figure 4:**
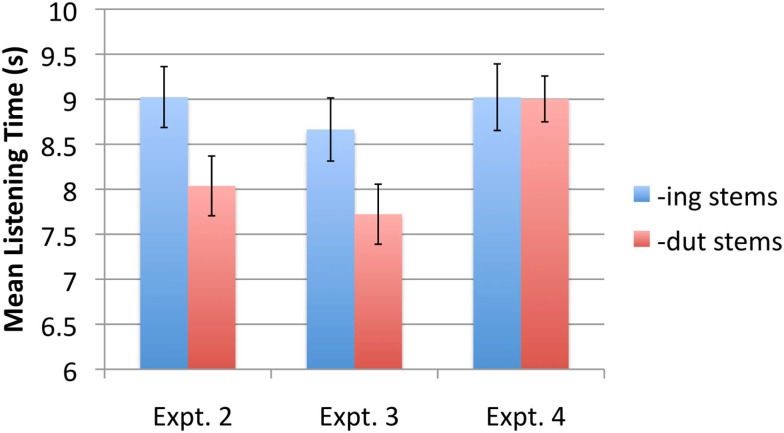
**Mean listening times to *-ing* stem and *-dut* stems in Experiments 2–4**.

The data were first submitted to a 2 × 2 × 2 ANOVA with stem type (*-ing* or *-dut*) and stem stress pattern (trochaic or iambic) as within-subjects factors, and counterbalance group (A or B) as a between subjects factor. Since the group variable did not interact with any other variable, data from the two groups were combined in subsequent analyses, to increase power. A 2 × 2 ANOVA was performed, with stem type (*-ing* or *-dut*) and stress pattern (trochaic or iambic) as within-subject variables. As predicted, there was a main effect of stem type, with infants listening on average for 9.02 s (SE = 0.34) to *-ing* stems compared to 8.04 s (SE = 0.33) to *-dut* stems [*F*(1,29) = 5.30, *p* = 0.029, $\eta_{p}^{2}$ = 0.154]^2^. Twenty two out of the 30 infants showed this pattern. There was no other significant main effect or interaction. Figure 4 graphs the mean listening times to *-ing* stems and *-dut* stems.

As in Experiment 1, infants responded differently to stems to which they were familiarized in words that ended in the English suffix, *-ing*, compared to stems to which they were familiarized in words that ended in a pseudo-suffix. However, here infants listening longer to *-ing* stems compared to the pseudo-suffix stems, whereas in Experiment 1 infants listened longer to the pseudo-suffix stems. The preference for familiarity here vs. novelty in Experiment 1 is not surprising when one considers the differences in design across the two experiments. In Experiment 1, infants were familiarized to the inflected words in isolation, whereas in this experiment, the words were embedded in English sentences. It is a reasonable assumption that 15-month-olds processed the additional rich structure in the familiarization input to some degree – segmenting words (Aslin et al., 1998), categorizing words (Höhle et al., 2004; Gerken et al., 2005; Mintz, 2006; Shi and Melançon, 2010), and accessing word meanings. Stimulus complexity has been proposed as an important influence on infants’ preference for novelty or familiarity in experimental paradigms such as the HPP: Higher complexity during familiarization and learning phases is associated with a preference for more familiar test material, as long as that complexity is within the domain of what infants can process and represent (Hunter et al., 1983; Hunter and Ames, 1988; Kidd et al., 2012). Hence, the increase in complexity and variety in the familiarization material from Experiment 1 to Experiment 2 is consistent with a shift from a novelty preference in Experiment 1 to a familiarity preference in Experiment 2.

The results of Experiment 2 thus provide further support for the hypothesis that 15-month-olds treat the suffix *-ing* as a distinct element. Experiments 1 and 2 compared sub-lexical segmentation with *-ing* and two different pseudo-suffixes. In both cases, the results indicated that infants segmented stems and endings differently when the ending was the English suffix vs. the non-English pseudo-suffixes.

In Experiment 1, however, the segmentation differences were only found for bisyllabic stems. Infants did not show evidence of a different pattern of sub-lexical segmentation with monosyllabic stems. One explanation was that when listening to a list of isolated words, the trisyllabic words (with bisyllabic stems) may have stood out against a background of mono- and bisyllabic words, and captured infants attention more than the bisyllabic words. In contrast to the relatively unnatural familiarization scenario in Experiment 1 (a long list of isolated words), Experiment 2 exposed infants to the novel words in a much more natural context, which might more fully engage language processing mechanisms and in turn facilitate the detection of familiar suffixes in bisyllabic words. Experiment 3 tests this prediction by exposing 15-month-olds to bisyllabic nonsense words in an experimental design that is similar to Experiment 2.

## Experiment 3

### Materials and methods

#### Subjects

Subject recruitment procedures were identical to those used in the previous experiments.

Data for 34 infants averaging 15 months of age (mean age 15 months 1 day, range 14 months 13 days to 15 months 14 days) were analyzed. Data from 19 additional infants were excluded due to failure to complete the experiment (13), excessive fussiness (3), parental interference (2), and experimenter error (1).

#### Stimuli and design

The familiarization and test stimuli were prepared in the same manner as in Experiment 2. The structure of the familiarization material conformed to the structure in Experiment 2, except the nonce words were bisyllabic rather than trisyllabic, and the stress pattern for all nonce words was trochaic. As in Experiment 2, there were two counterbalanced familiarization sets, A and B, such that the *-ing* stems inset A were the *-dut* stems inset B, and vice versa. The familiarization items for set A are given in Table 6. The test items were the four nonce stems alone: *fem, gorp, sib*, and *riz*. *Fem*, and *gorp* were *-ing* stems for group A subjects, and *-dut* stems for group B subjects. Likewise, *sib* and *riz*, were *-ing* stems for group B subjects, but *-dut* stems for group A subjects.

**Table 6:** **Familiarization sentences for subjects in group A, in Experiment 3**.

Sentences With *-ing* Words	Sentences with *-dut* Words
I see you *feming*!	Does Sam want to go *sibdut*?
Johny likes *gorping*!	I want to go *rizdut*!
Do you want to go *feming*?	Harold likes *sibdut*!
Can you see me *gorping*?	Can you see Sally *rizdut*?

*Familiarization sentences for subjects in group B were identical, except that *-ing* stems and *-dut* stems were switched*.

#### Procedure

The procedure was identical to the procedure in Experiment 2, except that there were seven, rather than six familiarization blocks. This is because the familiarization sentences were slightly shorter in duration, and the total duration of the familiarization period was kept to approximately 90 s.

### Results and discussion

As in the prior studies, test trials with orientation times under 1 s were replaced with the subject’s orientation time for the same stimulus in the other block. Data for three *-ing* stem trials were modified in this way.

For each subject, a mean orientation time for *-ing* stems was calculated by averaging orientation times to all *-ing* stem trials, across test blocks. An average orientation times to *-dut* stems was calculated in the analogous way, resulting in two data points per subject.

Subjects in the A and B familiarization groups did not differ in their overall response patterns to *-ing* vs. *-dut* stems [*t*(32) = 1.33, *p* = 0.19], so scores for the two groups were pooled. As in Experiment 2, infants listened significantly longer to *-ing* stems compared to *-dut* stems. Mean listening times were 8.7 s (SE = 0.35) and 7.7 s (SE = 0.334) for *-ing* and *-dut* stems, respectively [*t*(33) = 2.34, *p* = 0.026 two-tailed, *d* = 0.47]. Twenty two out of the 34 infants showed this pattern. Figure 4 graphs the mean listening times to the two stem types.

Infants thus behaved similarly here when tested on monosyllabic stems as they did in Experiment 2 when tested on bisyllabic stems: They listened reliably longer to the *-ing* stems compared to the *-dut* stems. Thus, as in Experiments 1 and 2, infants segmented stems out of familiarized words that had the English suffix, but not stems that carried the pseudo-suffix. Here, however, infants showed this segmentation difference for bisyllabic words, whereas in Experiment 1 they did not. As discussed earlier, the structure of the familiarization material could have focused infants’ attention on the more distinctive trisyllabic words, so that they were less likely to detect and segment *-ing* from monosyllabic stems. In addition, familiarizing infants to the nonce words in otherwise normal English sentences may have resulted in a greater engagement and activation of normal language processing mechanisms and representations, including the processing of familiar affixes such as *-ing*.

This experiment thus lends further support to the hypothesis that 15-month-old English-learners treat the English suffix *-ing* in a privileged way when processing speech. These findings are in accord with those of Marquis and Shi (2012), who showed that infants learning French represent elements of bound morphology by as early as 11 months. Marquis and Shi suggested that infants form distinct representations of bound morphemes, at least initially, simply because the forms are very frequent in their input. This explanation may be sufficient to account for the difference in segmentation between *-ing* and the pseudo-suffixes used here. However, a mechanism that considers the internal predictability of forms, perhaps in addition to their frequency, is also consistent with the present findings. For example, the word segmentation mechanism proposed by Saffran et al. (1996) segments sequences at junctures of low transitional probability between syllables. Sequences with high transitional probability may also be relatively high in frequency, but two sequences could be equal in frequency yet differ in internal transitional probabilities. Infants as young as 8-months appear to be sensitive to transitional probabilities, not just frequency (Aslin et al., 1998).

The functional similarity between word segmentation and the sub-lexical segmentation of bound morphemes – that is, extracting predictable sequences from larger sequences – could be mirrored by similarities in processing mechanisms. Since 8-month-old infants show evidence of statistically based word segmentation, it is thus possible that they also can detect highly regular patterns *within* words. Experiment 4 investigates this question by replicating the procedures and design of Experiment 2, but testing 8-month-old infants.

## Experiment 4

### Materials and methods

#### Subjects

Subject recruitment procedures were identical to those used in the previous experiments.

Thirty-six infants averaging 8 months of age (mean age 8 months 3 days, range 7 months 18 days to 8 months 20 days) were tested. Infants were randomly assigned to one of two familiarization groups, A or B, consisting of 18 infants each. Data from all 36 infants were analyzed.

#### Stimuli and design

The stimuli and design of the experiment was identical to Experiment 2.

#### Procedure and apparatus

The apparatus and testing procedure was identical to Experiment 2.

### Results and discussion

As in the prior experiments, any test trial with an orientation times under 1 s was replaced with the subject’s orientation time for the same stimulus in the other block. Data for one*-ing* stem test trial was modified in this way.

The data were first submitted to a 2 × 2 × 2 ANOVA with stem type (*-ing* or *-dut*) and stem stress pattern (trochaic or iambic) as within-subjects factors, and counterbalance group (A or B) as a between subjects factor. Since the group variable did not interact with any other variable, data from the two groups were combined to increase power. A 2 × 2 ANOVA was performed, with stem type (*-ing* or *-dut*) and stress pattern (trochaic or iambic) as within-subject variables. Neither main effect was significant, nor was the interaction (all *F*s < 1). As shown in Figure 4, infants’ listening times to *-ing* and *-dut* stems was 9.0 s (SE = 0.37) and 9.0 s (SE = 0.26), respectively.

Unlike in the previous experiments with 15-month-olds, there was no evidence that 8-month-olds treated *-ing* in a special way when processing the familiarization material. In principle, the mechanisms that are engaged in laboratory demonstrations of word segmentation in 7.5–8-month-olds could segment predictable sub-lexical patterns such as bound morphemes. However, this experiment provides no evidence that 8-month-olds are carrying out these kind of analyses. Of course, the design of the experiment assesses segmentation of suffixes indirectly, by measuring infants’ responses to stems. It could be that infants segmented *-ing* (but not *-dut*) during familiarization, but did not have sufficient exposure to the resulting stems to be able to recognize them during the test phase. Compared to word segmentation experiments, infants’ exposure to individual test items is much less in the experiments reported here. For example, in Saffran et al.’s (1996) study, infants were tested on words they had heard 45 times. The number of exposures in the present study may have been sufficient for 15-month-olds, but not for 8-month-olds. On the other hand, it also is possible that 8-month-olds have not yet begun to form long-term representations of sub-lexical forms.

The design of this experiment could be modified to increase exposure to nonce words. However, this runs the risk of providing infants with distributional evidence that the pseudo-affixes are also affixes, and infants may then start segmenting pseudo-affixes as well. Indeed, in one experiment, Marquis and Shi (2012) demonstrated that with sufficient exposure to a pseudo-suffix, /u/, French-learning 11-month-olds started treating the ending similarly to the actual French suffix, /e/, in their experimental task.

## General Discussion

Taken together, the experiments in this study demonstrate that English-learning 15-month-olds represent the suffix *-ing* as a discrete unit. Thus, although previous experiments failed to find evidence that 15-month-olds have acquired morphosyntactic dependencies involving *-ing* (Santelmann and Jusczyk, 1998), infants may nevertheless be in the process of learning these dependencies at this age. Specifically, having a discrete representation of an affix allows infants to notice dependencies between that affix and other forms.

It is important to note that while this study supports the hypothesis that infants treat *-ing* as a discrete entity at 15 months, it would be premature to conclude that they have acquired the form *qua* suffix of English. That is, there is no evidence that these forms are fully morphological, in the sense that infants represent them as elements that participate in dependencies and that are associated with certain semantic properties. (Indeed, Santelmann and Juscyk’s results suggest that infants have not yet learned basic patterns and dependencies involving *-ing*.) Initially, infants might represent bound morphemes as distinct entities simply by virtue of the fact that they occur frequently within words, as suggested by Marquis and Shi (2012). The results from the present study are entirely consistent with that proposal. In an examination of the input to the child Peter, in the Bloom corpus (Bloom et al., 1974, 1975) of the CHILDES database (MacWhinney, 2000), 2.2% of word tokens and 6.9% of word types spoken by adults to Peter ended in /Iŋ/ (regardless of whether the ending was a morpheme or not, as in *sing*). In contrast, only 0.6% of tokens and 0.5% of word types ended in /ɑt/, and there were no words that ended in the sequence /dʌt/^3^.

Although Marquis and Shi (2012) discuss infants’ early representations of bound morphemes in terms of the frequency of sub-lexical patterns, it is reasonable to conjecture that the detection of sub-lexical forms may also depend on transitional probabilities. That is, when a frequent form occurs in many different contexts, it might be more likely to be identified as a distinct form than a form of equal frequency that occurs in a more restricted set of contexts. The mechanisms for segmenting sub-lexical forms would then be computationally similar to mechanisms that have been proposed for detecting words in fluent speech (Saffran et al., 1996; Aslin et al., 1998; Pelucchi et al., 2009). While this may be so, Experiment 4 did not find evidence that 8-month-olds detected and segmented *-ing* from nonsense words, although infants had relatively few exposures to the novel forms compared to other experiments in word segmentation. Future research, using different methods, can further probe how early infants start to segment and represent bound morphemes as distinct forms.

Beyond distributional properties such as frequency and transitional probabilities, phonological factors could also influence infants’ early representation of affixes. To the degree that affixes in a given language have phonotactic tendencies that infants can detect, once infants have segmented enough affixes to detect the patterns, they could use the tendencies as cues to guide further segmentation and the discovery of new affixes. This possibility raises a potential concern in this study: Although, as just reported, the frequency of /ɑt/ and /dʌt/ at the ends of words in children’s input is very low or virtually absent, the two pseudo-affixes are not parallel in comparison to real English affixes when analyzed at a more general level. Specifically, no English inflectional suffix has a CVC structure, like /dʌt/ (although some derivational affixes do, e.g., *-tion*), but there are frequent affixes with a VC structure, like /ɑt/ (e.g., /Iŋ/, /әz/, /әd/). In principle, if infants are sensitive to these broader phonotactic properties of English inflectional affixes, the atypical structure of *-dut* could have caused infants to reject *-dut* as a possible suffix in Experiments 2 and 3.^4^ This possibility offers another explanation for the differing results with respect to monosyllabic stems in Experiment 1 compared to Experiment 3: Infants may be relatively more likely to treat *-ot* as a possible suffix because of its phonological structure, and given the simpler overall structure of bisyllabic words, segmented both *-ing* and *-ot* from the shorter words in Experiment 1. Of course, this study was not designed to test these broader generalizations of phonological form. Nevertheless, to address this possibility, a followup study with adults was carried out; the experiment was designed to assess whether experienced English users show an advantage in segmenting *-ot* – which conforms to English inflection structure – from nonce word forms, compared to *-dut*, which does not. Fifteen native English speakers listened to the same nonce words that ended in -*dut* and -*ot* that were used in these studies, but the words were presented in a rapid sequence, with 1.1 s between word onsets. From time to time, two words in a row both ended in *-dut* or both in *-ot*. Participants had to press a key whenever they heard a word that rhymed with the word before it. The question of interest was whether participants differed in their accuracy in detecting rhymes with *-ot* compared to rhymes with *-dut*. A logistic regression with the ending (*-dut* vs. *-ot*) as a within-subjects variable did not reveal any difference in accuracy in detecting rhymes with *-dut* (on average 78% detected) compared to rhymes with *-ot* (on average 68% detected; *p* = 0.336). So for adults, there is apparently no advantage for one form or the other with respect to ease of detection. Interestingly, there was a slight reaction time advantage for *-dut* rhymes (607 ms, measured from suffix onset) compared to *-ot* rhymes [653 ms; *t*(14) = 2.20, *p* < 0.05]. Although these findings from adults are hardly conclusive concerning infants’ knowledge of inflections, they at least suggest that infants would not be biased against segmenting *-dut* compared to *-ot* from pseudo-stems, despite the fact that the former is atypical with respect to inflectional suffixes in English.

The modest but reliable speed advantage for detecting *-dut* over *-ot* in adults could be related to the fact that -*dut* is a complete syllable, whereas *ot* lacks an onset and is subject to resyllabification with segments at the end of the stem. Indeed, the motivating factor for using -*dut* in Experiments 2–4 was to use a pseudo-affix that was relatively easy to segment on structural grounds, thus providing a stronger test of infants’ treatment of *-ing* as a privileged form. However, going beyond the methodological considerations of this study, perceptual factors relating to affix syllable structure is another way in which phonological variables could play a role in infants’ acquisition of affixes: All else being equal, affixes that are subject to resyllabification might be harder to detect and take longer to acquire than affixes that are not. Cross-linguistically, there is some support for this notion. For example, Turkish morphemes are generally syllabic and contain unreduced vowels, and many have onsets. Children learning Turkish show productive use of morphemes somewhat in advance of children learning English (Aksu Koç and Ketrez, 2003). In the present study, although *-ing* lacks an onset, it stands out from most other inflectional morphemes in English in that it has a full vowel. It is also typically the first inflectional morpheme to be reliably produced when required by children learning English. It is possible, then, that while 15-month-olds have identified this “robust” morpheme as a distinct form, they have not yet formed independent representations of other English morphemes. Exploring this question by testing different morphemes will clarify the role of the perceptual properties of suffixes that may influence how bound morphemes are first represented.

Finally, in addition to the potential role of frequency in infants’ acquisition of affixes (Marquis and Shi, 2012), more general distributional properties of a language’s inflectional system may influence infants’ detection of bound morphemes. As alluded to earlier, one might expect the developmental timing of the first representations of morphemes to depend on the richness of a language’s overt morphological marking. Learners of languages with rich morphological marking (such as French) may begin to detect and represent sub-lexical forms in advance of their peers learning languages that are morphologically more “impoverished” (such as English). The acquisition of Turkish, again, provides some evidence for this view. Turkish makes extensive use of morphological marking, and children show productive use of morphemes as early as 17 months (Aksu Koç and Ketrez, 2003). However, such comparisons are complicated by the phonological and perceptual factors discussed earlier.

## Conclusion

A significant component of language, both in structure and in content, resides in the sub-lexical combinatorial units – the bound morphemes. In acquiring a language, learners must acquire the semantic and structural properties of bound morphemes, but before doing so, they must identify what the relevant sub-lexical units are in their language. The experiments reported here demonstrate that English-learning 15-month-olds represent *-ing* as a distinct form. When processing novel words that end in *-ing*, they segment the suffix from the stem. This allows them to notice morphosyntactic and morphosemantic patterns that involve that form, and that will form a part of their acquired grammatical knowledge. In addition, by representing word stems as distinct forms, infants can then detect morphosyntactic patterns involving the stem, such as other inflectional paradigms. Thus, at an age where many learners are not yet combining words in their own speech, and before they use bound morphemes productively, infants have started to develop representations of the morphology of their language.

## Conflict of Interest Statement

The author declares that the research was conducted in the absence of any commercial or financial relationships that could be construed as a potential conflict of interest.

## Footnotes

^1^I am grateful to an anonymous reviewer for suggesting this interpretation.

^2^In this and subsequent experiments, the number of subjects that contributed to the data analyses was greater than in Experiment 1. This is because we anticipated relatively high dropout rates based on piloting, so the research assistants were given a high quota for the number of subjects to test in a given experiment. As a result, we ended up with more subjects than in the initial study. However, restricting the data analysis to the first 12 subjects per counterbalance condition in Experiments 2–4 (as in Experiment 1) yields an identical pattern of results to the reported ones that include additional infants.

^3^The CMU pronouncing dictionary (http://www.speech.cs.cmu.edu/cgi-bin/cmudict) was used to identify orthographic forms corresponding to words that ended in the relevant phoneme sequences. The combined frequency of those forms was then tallied in the corpus of child-directed speech.

^4^This possibility was suggested by an anonymous reviewer.
